# LiDCOrapid and PiCCOplus preload response parameter validation study

**DOI:** 10.1186/cc9481

**Published:** 2011-03-11

**Authors:** P Brass, E Mills, J Latza, J Peters, E Berendes

**Affiliations:** 1 Helios Klinikum, Krefeld, Germany; 2 LiDCO Ltd, London, UK; 3 Klinikum Duisburg, Germany

## Introduction

This study compares the ability of two arterial waveform monitors, the calibrated PiCCOplus and the nomogram scaled LiDCOrapid, to detect fluid responsiveness using the functional hemodynamic parameters stroke volume variation (SVV) and pulse pressure variation (PPV) in a surgical ICU population (ventilated, closed chest). The passive leg raising test (PLRT) is an alternative reversible test that can be carried out before administering volume.

## Methods

We recruited 20 patients who had undergone major abdominal or neurosurgery and 10 patients in the SICU with progressive circulatory instability. The femoral artery was cannulated to obtain the arterial blood pressure waveform. Simultaneous measurements were made at four time points, M1 to M4: (M1) baseline, (M2) after PLRT, (M3) baseline (M4), after 500 ml Tetraspan^® ^6% over 10 minutes via pressure infusion. The PiCCO was calibrated via transpulmonary thermodilution at each time point. A change in SV >10% was considered as volume responsive.

## Results

**Figure 1:**
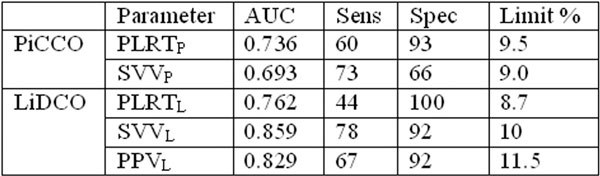


Data were collected from 30 patients, age 31 to 90, ASA 2 (2), ASA 3 (24) or ASA 4 (4), BSA 1.54 to 2.52 m^2^. Patients were ventilated with at least 6 ml/kg (IBW), and RR of 10 to 15/minute. PiCCO identified 15 patients as responders (50%) and LiDCO identified 18 patients as responders (60%) to the fluid challenge, both within the normal range of established studies. ROC curve analysis results are shown in Figure 1. Bland-Altman analysis comparing the PPVL with SVVL and SVVP give a bias of 0.3% and 0%, and limits of agreement of ± 3.8% and ± 4.4%, respectively.

## Conclusions

This study has demonstrated that SVV, PPV, and PLRT to a lesser extent, are effective for predicting volume response and can be used perioperatively for fluid management as part of goal-directed therapy. The sensitivity and specificity of the SVVL and PPVL were both greater than the SVVP. This is probably due to the difference in each algorithm's ability to identify responders to the fluid challenge.

